# Transthyretin Binding Mode Dichotomy of Fluorescent *trans*-Stilbene Ligands

**DOI:** 10.1021/acschemneuro.2c00700

**Published:** 2023-02-13

**Authors:** Afshan Begum, Jun Zhang, Dean Derbyshire, Xiongyu Wu, Peter Konradsson, Per Hammarström, Eleonore von Castelmur

**Affiliations:** Linköping University, IFM-Department of Physics, Chemistry and Biology, 58183 Linköping, Sweden

**Keywords:** Transthyretin (TTR), amyloidosis, crystal structure, ligand, fibrillation inhibitor, fluorescence

## Abstract

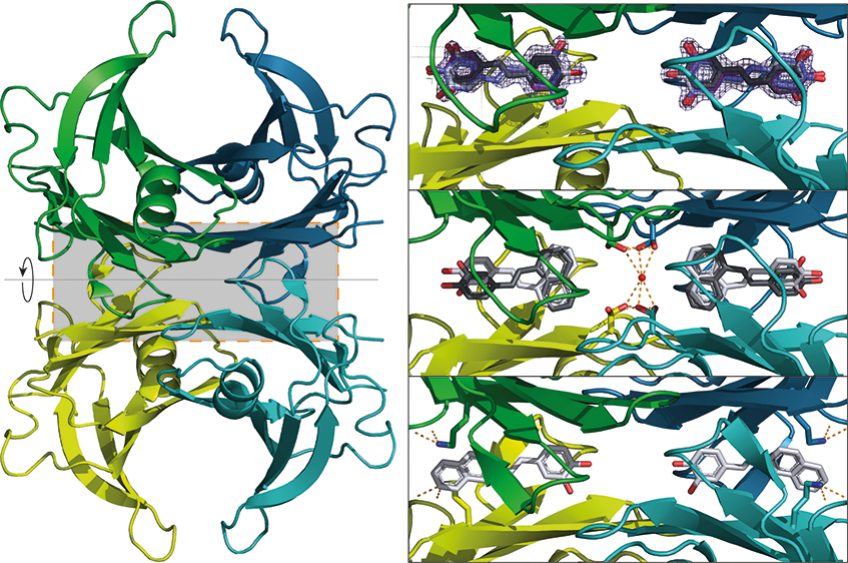

The orientations of ligands bound to the transthyretin (TTR) thyroxine (T4) binding site are difficult to predict. Conflicting binding modes of resveratrol have been reported. We previously reported two resveratrol based *trans*-stilbene fluorescent ligands, (*E*)-4-(2-(naphthalen-1-yl)vinyl)benzene-1,2-diol (SB-11) and (*E*)-4-(2-(naphthalen-2-yl)vinyl)benzene-1,2-diol (SB-14), that bind native and misfolded protofibrillar TTR. The binding orientations of these two analogous ligands to native tetrameric TTR were predicted to be opposite. Herein we report the crystal structures of these TTR:ligand complexes. Opposite binding modes were verified but were different than predicted. The reverse binding mode (SB-14) placing the naphthalene moiety toward the opening of the binding pocket renders the fluorescent ligand pH sensitive due to changes in Lys15 amine protonation. Conversely, the forward binding mode (SB-11) placing the naphthalene inward mediates a stabilizing conformational change, allowing intersubunit H-bonding between Ser117 of different monomers across the dimer interface. Our structures of TTR complexes answer important questions in ligand design and interpretation of *trans*-stilbene binding modes to the TTR T4 binding site.

## Introduction

### TTR in Transport, Chaperone and Amyloid Processes

Transthyretin (TTR) is a plasma and cerebrospinal fluid (CSF) protein synthesized by the liver, choroid plexus, eye and pancreas. TTR is the main transporter of T4 hormone in CSF and the primary carrier of retinol (Vitamin A) binding protein in blood plasma. TTR appears to function as a molecular chaperone in preventing amyloid formation of Aβ in the brain^1−4^ and islet amyloid polypeptide (IAPP) in the pancreas.^5^ TTR is inherently amyloidogenic, and the wild-type protein accounts for a significant number of cardiac amyloidosis cases in elderly people^6^ as well as diverse mutation-dependent amyloid disease phenotypes in familial diseases such as familial amyloid polyneuropathy (FAP).^7^ Collectively this group of diseases are known as transthyretin amyloidosis with deposited ATTR. TTR research to understand protein misfolding diseases is of considerable interest due to the impact of TTR amyloidosis on the afflicted ATTR patient and society. The small molecule ligand Tafamidis is the active component of Vyndaqel and Vyndamax, which are approved drugs for treating FAP and wild-type ATTR cardiomyopathy. Tafamidis works as a kinetic stabilizer by binding to the T4 binding site, preventing tetramer dissociation, and thereby subsequent misfolding and fibril formation is suppressed.^8^ Tafamidis binding also imposes long-range allosteric conformational changes^9^ which may in addition to preventing tetramer dissociation inhibit aberrant proteolysis, which is a putative fibrillation initiation mechanism.^10^

### Resveratrol Is a Health Promoting Natural Compound

*trans*-Resveratrol is a *trans*-stilbene polyphenolic antioxidant found predominantly in plants such as grapes. Resveratrol can reach significant concentrations in red wine. There are reports of resveratrol being anti-inflammatory, anti-cancer, neuroprotective, and anti-aging, similar to effects of calorie restriction.^11^ The latter activity is largely attributed to resveratrol isoform selective activation and inhibition of the deacetylase activity of sirtuins.^12^ The class of *trans*-stilbenes have been of considerable interest for TTR amyloidosis to find natural products as alternatives to kinetic stabilizers of TTR tetramers such as tafamidis.^8^

### *trans*-Stilbene Ligands for Amyloid Proteins

TTR is a homo tetramer with a total mass of 55 kDa composed of 127 amino acids in each subunit. The structure of TTR was originally solved by Blake in 1978,^13^ and numerous structures of TTR in complex with ligands have been published since. With all this knowledge there has been controversy regarding certain binding modes of ligands to TTR. Studies of TTR-targeting molecules are of significant interest due to TTR association with age-dependent amyloidosis. TTR is a rather promiscuous binder of aromatic compounds resembling T4 in its dual T4 binding pockets. Resveratrol has been reported to bind to the T4 binding pocket (T4BP) with rather high affinity. Interestingly, it appears that the binding modes of several molecules to TTR are also promiscuous regarding preferred binding orientation. Crystal structures of the TTR:resveratrol complex report contrasting binding modes.^14,15^

That resveratrol can be used as a TTR tetramer-sensitive fluorescent ligand when binding to the T4BP was shown many years ago.^16^ Furthermore, the *trans*-stilbene chemical motif is of particular interest for amyloid targeting because it is present in several amyloid fibril-specific ligands including fluorescent ligands X-34, Methoxy-XO4, and PET ligands florbetaben (18F) and florbetapir (18F).^17^ Our rationale for the current study is of general interest for understanding the selectivity and binding modes of TTR-binding *trans*-stilbene ligands and in particular for resveratrol and resveratrol analogues of amyloid fibril probes.

We previously hypothesized on the binding mode of two structurally homologous, amyloid-sensitive ligands SB-11 and SB-14.^18^ Based on fluorescence spectroscopy, we speculated that they display opposite binding orientations in the T4BP of TTR.^18^ We determined the structures of these compounds bound to TTR by X-ray crystallography to verify these findings. Here we present the crystal structures of wild-type TTR in complex with three *trans*-stilbene compounds TTR:SB-11, TTR:SB-14, and TTR:resveratrol. The data confirm our previous findings that these ligands bind in opposite directions despite being analogues and explain their distinct activities in terms of fluorescence and as stabilizing inhibitors to prevent fibril formation.

## Results and Discussion

### Crystal Structures of TTR:Ligand Complexes

**Figure 1:**
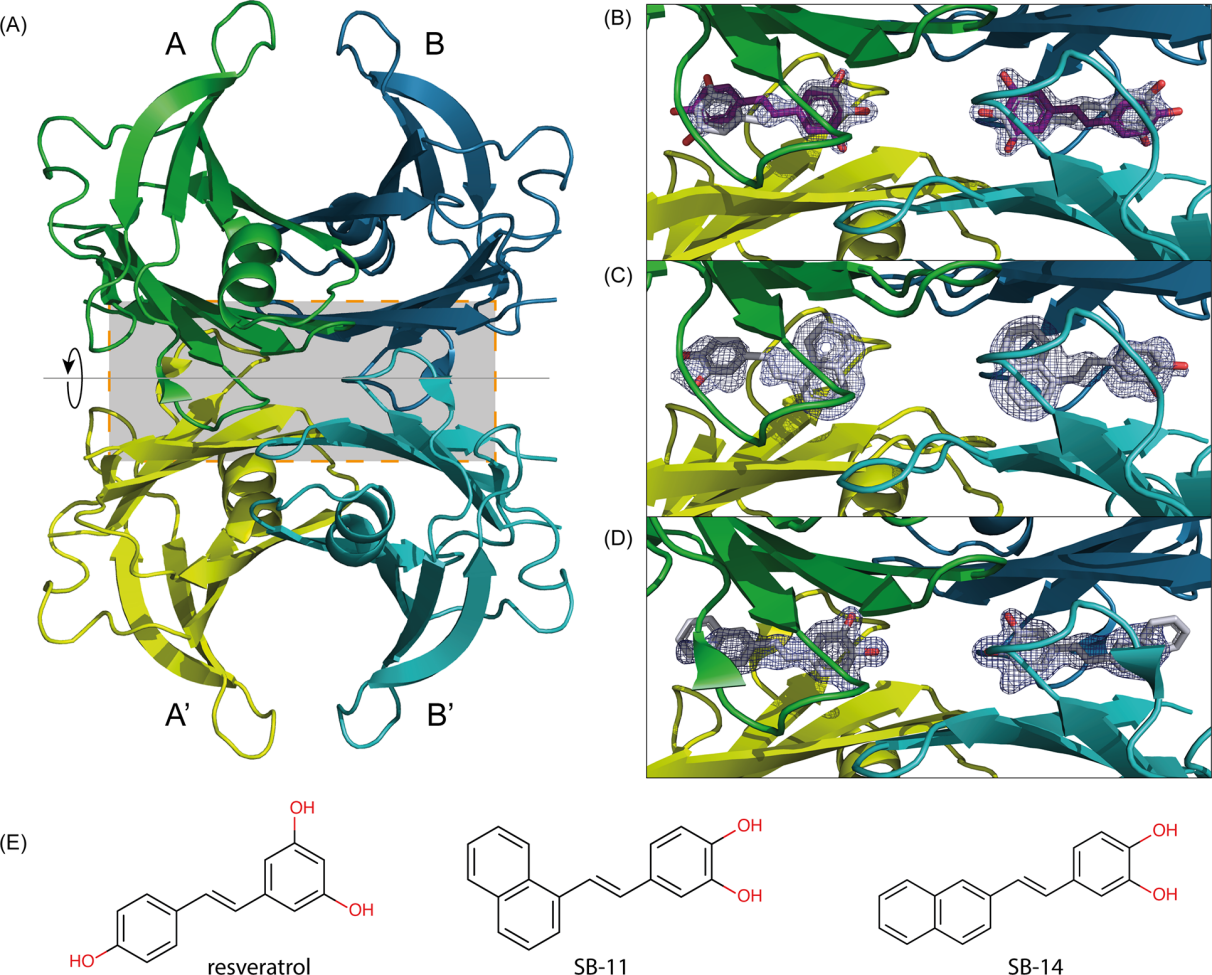
Electron density permits unambiguous placement of ligands in the TTR T4BP. (A) TTR is depicted as a cartoon, with monomer A in green and monomer B in petrol blue. Monomers A′ and B′ are colored light green and teal, respectively. The same coloring scheme for TTR is applied throughout all figures. For the complex structures with (B) resveratrol, (C) SB-11, and (D) SB-14, ligands are drawn as sticks and the 2mFo-DFc electron density contoured at 1.5 sigma is shown as blue mesh. (E) Chemical structures of the ligands. .

High-resolution X-ray crystal structures of TTR in complex with SB-11, SB-14, and resveratrol were obtained by cocrystallization with ligands coincubated with the protein for at least 2 h at room temperature. Comparisons were done with apo-TTR crystallized under identical conditions. All four crystal structures belong to the *P*2_1_22_1_ space group; the protein structure remains unchanged upon ligand binding, as evidenced by the low deviation in Cα positions between the aligned monomers (<0.35 Å rmsd). The dimer AB is found in the asymmetric unit and the second dimer (A′B′) to form the tetramer can be obtained by rotation along the crystallographic 2-fold *c*-axis (Figure 1A). The inner β-sheets of the dimer–dimer (AB–A′B′) interface form two T4BP cavities referred to as sites AA′ and BB′, respectively. The symmetric binding sites within each AA′ and BB′ dimer are composed of three so-called halogen binding pockets (HBPs). HBP1 is in the outer cavity close to the protein surface and HPB3 is in the inner cavity closest to the center of the tetramer, with HPB2 between these two cavities. The T4BP is predominantly hydrophobic, though some of the constituent amino acids have polar side chains that allow hydrophilic interactions.

**Figure 2:**
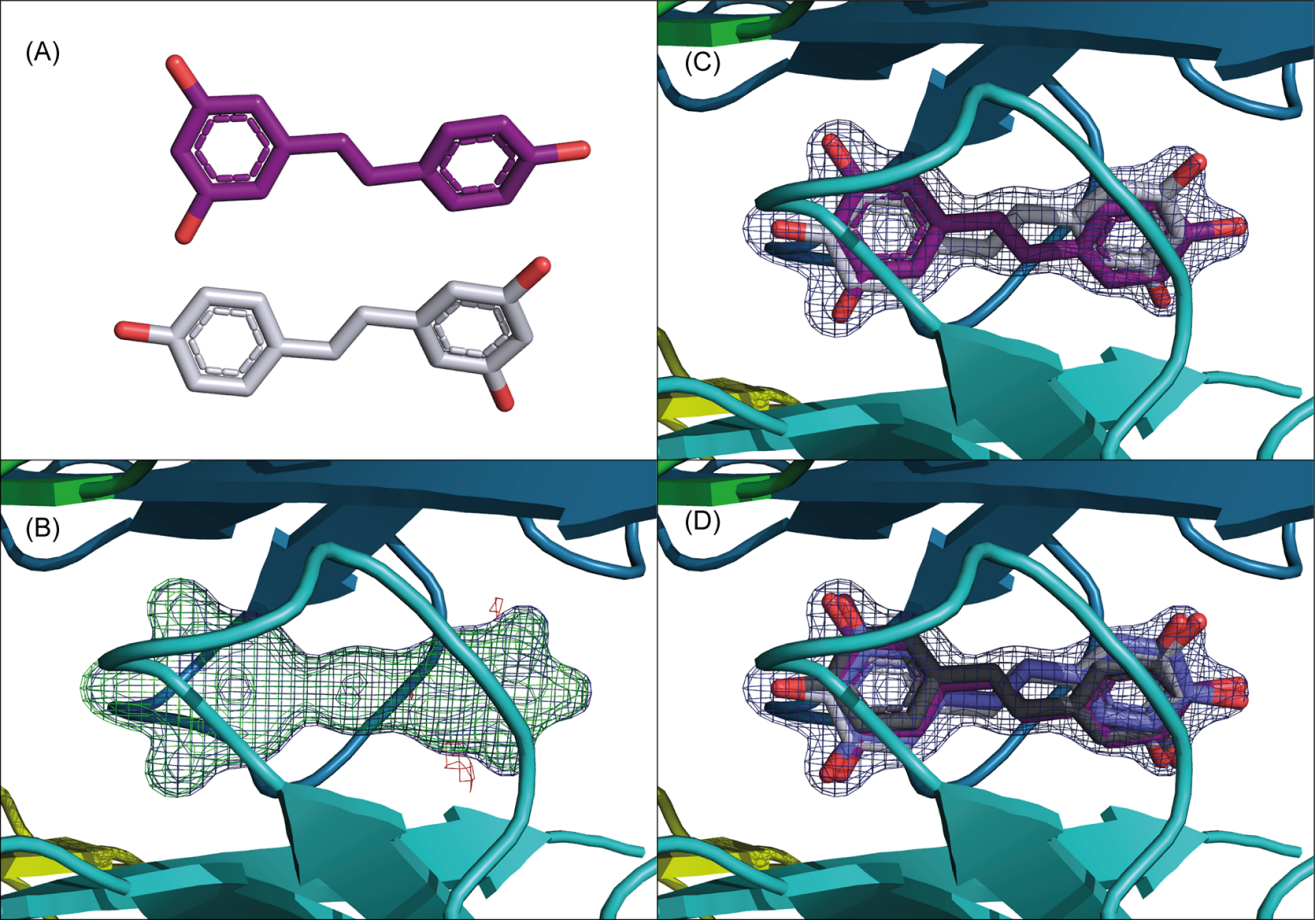
Resveratrol binds to TTR in both orientations. (A) Resveratrol can bind to the T4BP in two orientations. (B) The electron density in the BB′ T4BP prior to placement of the ligand supports both binding orientations of resveratrol. The 2mFo-Fc map contoured at 1σ is shown as blue mesh, and the mFo-DFc difference density contoured at 3.5σ is shown as green/red mesh. (C, D) Dual binding mode of resveratrol (C) observed in the asymmetric unit and (D) after applying the 2-fold symmetry creating the tetramer, showing both symmetry mates of the ligand. .

**Figure 3:**
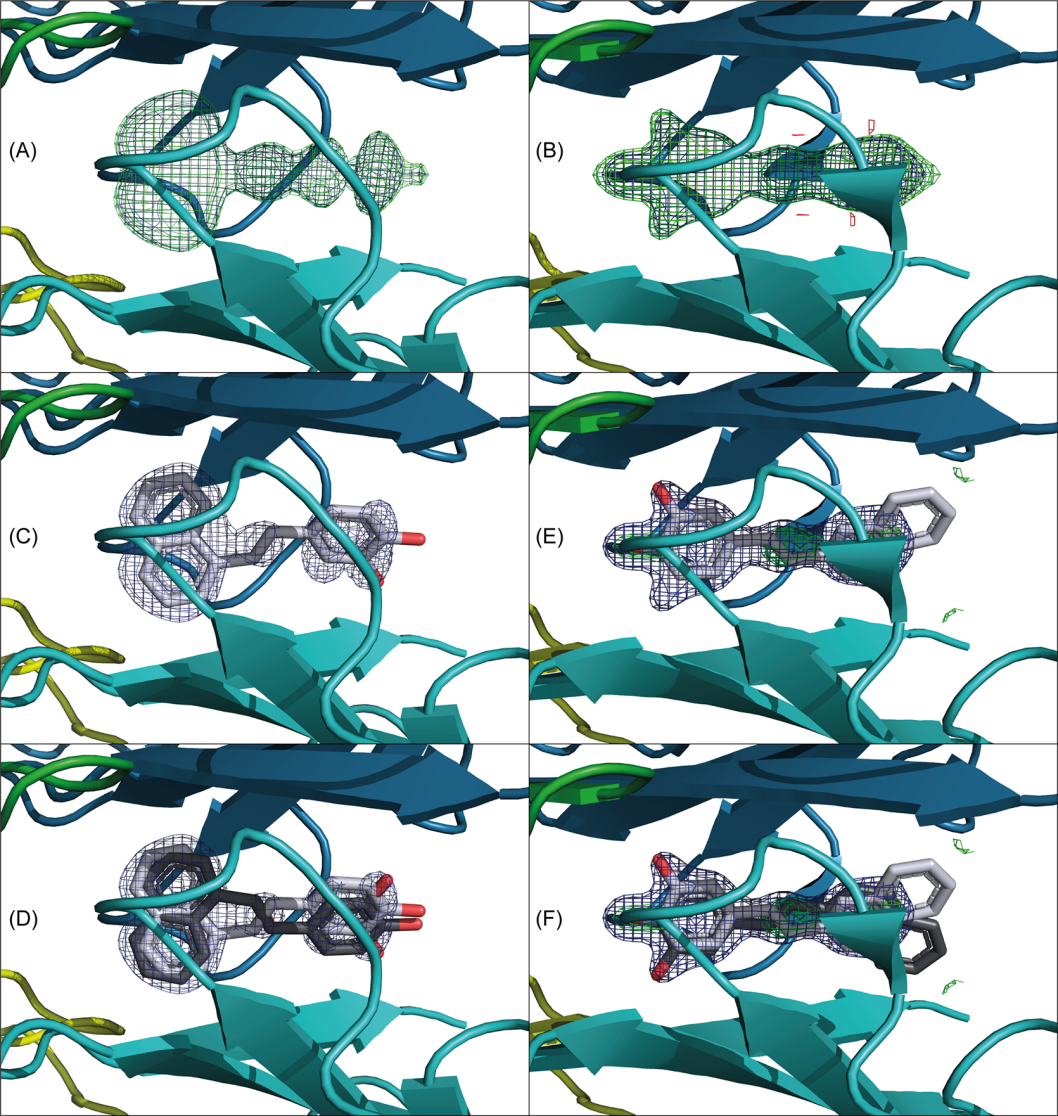
Structural analogues SB-11 and SB-14 bind to TTR in opposite orientations. (A, B) The electron density in the BB′ T4BP prior to ligand placement (A, SB-11; B, SB-14) is shown with 2mFo-Fc in blue mesh contoured at 1.5σ and mFo-Fc as green/red mesh contoured at 3.5σ. (C) SB-11 showing exclusively forward binding mode with the polar dihydroxy-benzene ring outside toward HBP1 and the opening of the T4BP while the naphthalene moiety sits inside HPB3. (D) Superposition of both symmetry mates of SB-11 (light/dark gray). (E) SB-14 showing exclusively reverse binding mode with the dihydroxy-benzene ring inside HPB3 of the T4BP and the naphthalene pointing toward HBP1. (F) Both symmetry mates of SB-14 are shown. Averaging of the density at the special position could explain the lack of electron density for the asymmetrically superposing naphthalene ring in this orientation. In panels C–F, the 2mFo-DFc electron density contoured at 1.5σ is shown as blue mesh and the mFo-DFc difference density contoured at 3σ in green (pos)/red (neg).

**Figure 4:**
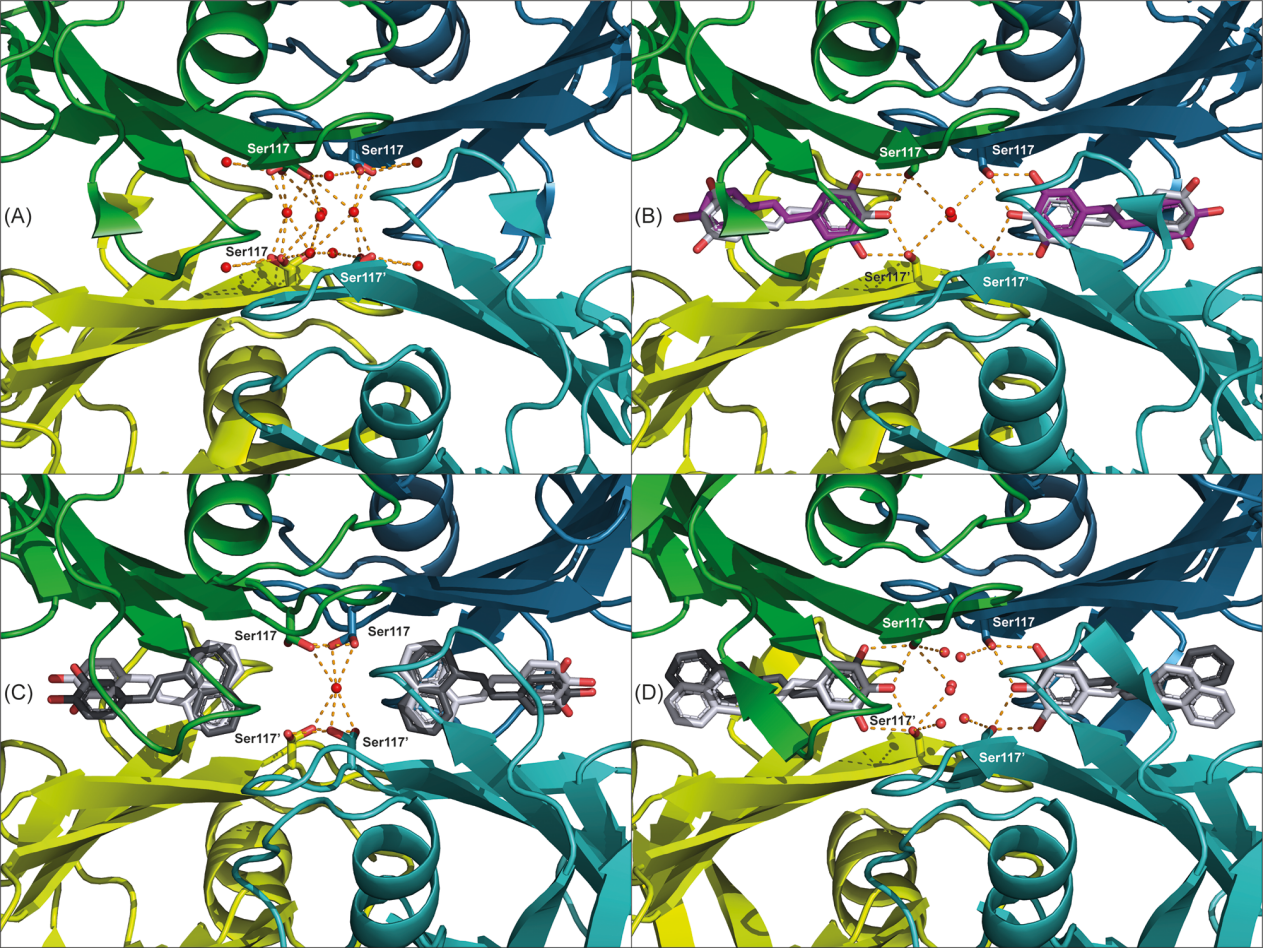
Ligand binding mode influences Ser117 conformational flexibility. Ordered water molecules within H-bonding distance of Ser117 are shown as red spheres, and H-bonds are shown as dashed orange lines. (A) Apo-TTR with fluctuating Ser117 extensively H-bonds to ordered water molecules within the binding pockets. (B) Resveratrol, regardless of pose, has hydroxyl groups within H-bonding distance of Ser117. Ser117 attains an outward conformation, hydrogen-bonding to the hydroxyl groups of resveratrol. (C) SB-11 showing exclusively forward binding mode with the naphthalene moiety inside HPB3 imposes an inward conformation of Ser117 rendering an internal H-bond between Ser117A–Ser117B across the dimer interface as well as an ordered water molecule in the tetramer core H-bonding to all four serines. (D) SB-14 showing exclusively the reverse binding mode with the polar dihydroxy-benzene ring within H-bonding distance of Ser117. Ser117 adopts an outward conformation H-bonding to the hydroxyl groups of SB-14 as well as ordered water.

Since the crystallographic 2-fold axis crosses the T4BPs, symmetry-related ligands are found superposed at this special position as already reported.^15,19^ The electron densities for all TTR:ligand complexes presented enabled unambiguous placement of ligands (Figure 1B–D, Figures 2–3). All ligands bind to TTR in the T4BP, but with different orientations. Ser117, in the innermost part of the binding pocket (HBP3), is known to adopt multiple conformations and can hydrogen-bond with the ligand (Figure 4). While SB-11 and SB-14 each have a single but distinct orientation, the observed electron density clearly supports both previously observed orientations for resveratrol (Figures 1–2).

### Structural Consequences of Different Binding Modes

Binding of resveratrol (in both orientations) and SB-14 with their hydroxyl groups pointing into HBP3 positions these within distance for H-bonding with Ser117 at the base of the T4BP (Figures 1–3). In comparison, SB-11 binds in the opposing forward mode, burying its hydrophobic naphthalene moiety in HBP3 (Figures 1, 3). Notably, in apo-TTR, the side chain of Ser117 adopts several alternative conformations and H-bonds with water molecules occupying the binding cavity (Figure 4A). Upon ligand binding, the conformation of Ser117 becomes more constrained. A common conformation for Ser117 is imposed when its hydroxyl group H-bonds with a polar ligand as observed for resveratrol (in either pose) and the asymmetric SB-14 (Figure 4B, D). In contrast, SB-11 induces intersubunit H-bonding interactions between two neighboring Ser117 (A:B and A′:B′) (Figure 4C). This latter pose is suggestive of a tetramer-stabilizing conformation with H-bonds across the dimer interface. Furthermore, in the SB-11 structure, a specific central water molecule appears to engage Ser117 by H-bonds from all four monomers across the dimer–dimer interface (Figure 4C).

### TTR Tetramer Stability and Inhibition of Fibril Formation

The tetramer-stabilizing activity of the ligands was assessed by differential scanning fluorimetry (DSF). At neutral pH TTR has a midpoint of thermal denaturation (*T*_m_) close to 100 °C and an increase in stability is therefore not easily assayed. Consequently, we selected pH 5.0 as the pH for this assay where the *T*_m_ for TTR is 92.3 °C. Resveratrol and SB-14 provided a rather modest +1.1 °C and +1.3 °C thermal shift, respectively. Interestingly, we observed that SB-11 markedly elevated the *T*_m_ more (+3.0 °C) than SB-14 (Table 1). In addition, in line with this thermal stabilization, SB-11 was a better fibrillation inhibitor at pH 4.4 than resveratrol and SB-14 (Table 1). The same trend of SB-11 activity outperforming resveratrol and SB-14 was also true for the TTR FAP mutation V30M both regarding thermal stability and fibril inhibition (Table 1). The stabilizing effect of SB-11 is consistent with the conformational rigidity of Ser117 and differences in the hydrogen-bonding networks observed when comparing our structures. Although the ligands in this study are not negatively charged at the pH of our experiments, the forward binding mode of SB-11 orienting the hydrophobic naphthalene toward HBP3 and polar groups toward the exposed Lys15 is consistent with previous structures for flufenamic acid,^20^*N*-phenyl phenoxazines,^21^ and Tafamidis.^8^

**Table 1:** Stability and Fibril Formation Inhibition

TTR sample	*T*_m_ (°C), pH 5.0	Δ*T*_m_ (°C)^b^	% Fibril, pH 4.4	% Fibril inhibition^c^
WT (vehicle)	92.3 ± 0.1	0	100	0
WT + Res^a^	93.4 ± 0.1	1.1	36 ± 4	64
WT + SB-14	93.6 ± 0.2	1.3	27 ± 13	73
WT + SB-11	95.3 ± 0.1	3.0	13 ± 0.3	87
V30M (vehicle)	81.0 ± 0.3	0	100	0
V30M + Res	82.0 ± 0.3	1.0	57 ± 11	43
V30M + SB-14	82.8 ± 0.3	1.8	29 ± 3	71
V30M + SB-11	82.9 ± 0.02	1.9	15 ± 6	85

a Res = resveratrol.

b Difference in midpoint of thermal denaturation, at pH 5.0, by DSF in the presence and absence of ligand (vehicle, 1% DMSO).

c Inhibition of fibril formation (turbidity at 400 nm), after 72 h of incubation at pH 4.4, calculated from the turbidity set to 100% in the absence of ligand (vehicle, 1% DMSO) compared to the presence of ligand.

### Naphthalene Positioning Governs the pH-Dependence of Fluorescence Properties

**Figure 5:**
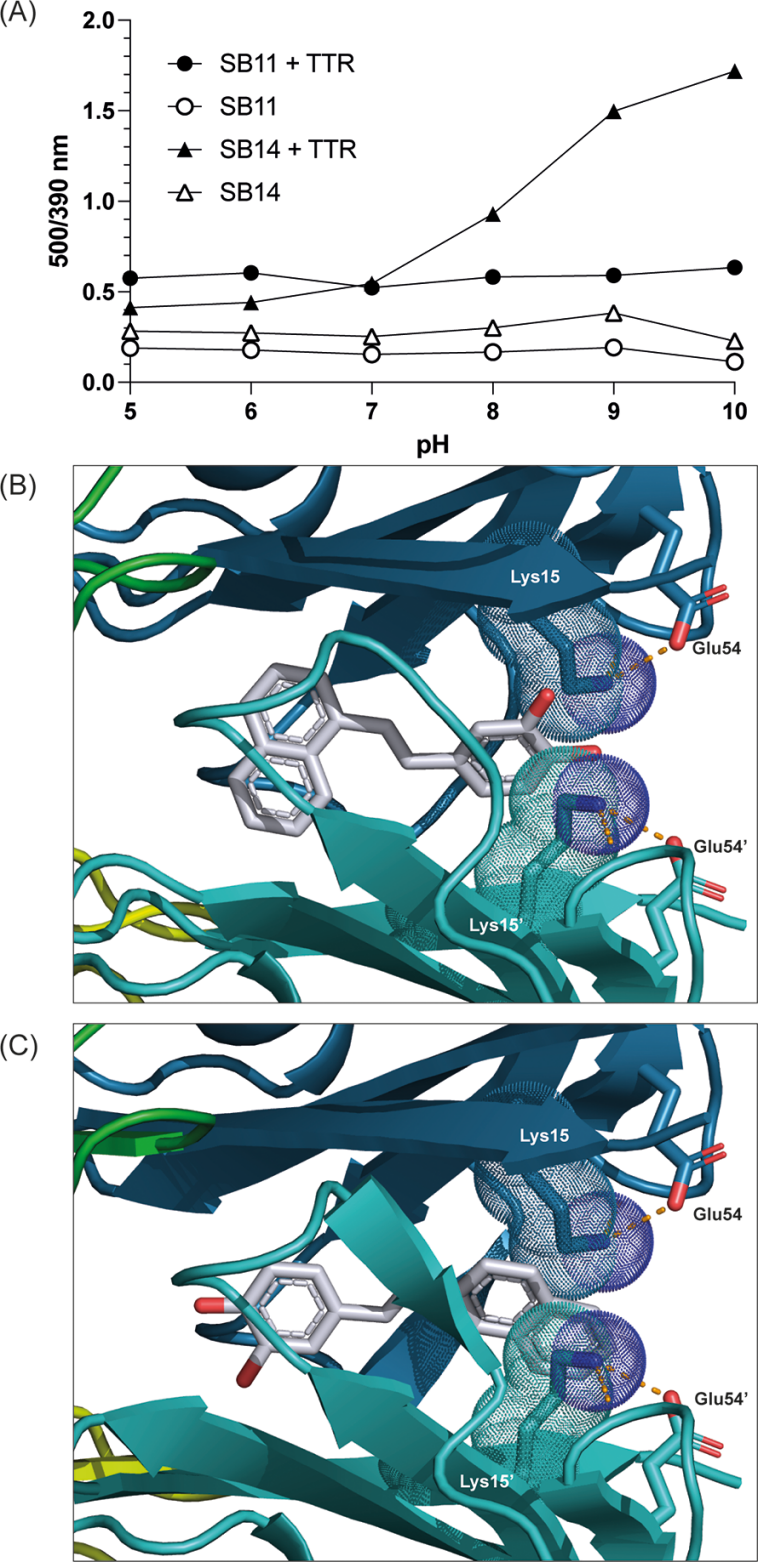
Fluorescence sensitivities of SB-11 and SB-14 toward pH are dependent on binding orientation. (A) Fluorescence spectra dependency on pH plotted as intensities of the 500 and 390 nm emissions (excitation at 350 nm). Ligands alone (open triangles and circles) are poorly fluorescent and are not pH sensitive. SB-14 bound to TTR (closed triangles) is highly sensitive with an apparent p*K*_a_ around 8.5. SB-11 is not sensitive to pH when bound to TTR (closed circles). (B) SB-11 with forward binding mode is insensitive to pH. (C) SB-14 with reverse binding mode positioning the naphthalene facing Lys15 at the opening of the T4BP.

That the binding mode appeared to affect ligand properties was evident from our previous pH-dependent fluorescence studies^18^ (Figure 5A). Interestingly, this property was limited to SB-14 when bound to TTR, showing a suppression of blue emission (390 nm) versus green emission (500 nm) illustrated in Figure 5A. Now, our structural data reveals that the reverse binding mode of SB-14 aligns the naphthalene moiety facing Lys15–Lys15′ at the entrance of the T4BP (Figure 5A). The pH sensitivity of SB-14 bound to TTR likely reflects the protonation state of the amine headgroup of Lys15 as described previously^18^ (apparent p*K*_a_ of 8.5) in proximity of the naphthalene moiety of SB-14 (Figure 5C). SB-11’s fluorescence ratio was instead rather unaffected by pH in the range pH 5–10 (Figure 5A). The binding mode observed in the TTR:SB-11 structure positions the naphthalene moiety inside HBP3, now explaining its reduced pH sensitivity (Figure 5B). While the different positioning explains the different pH sensitivity of SB-14 versus SB-11, it does not explain the green fluorescence (500 nm) which interestingly was unique for TTR-T4BP binding for both ligands compared to fibril binding.^18^

## Conclusions

Misfolding and aggregation of TTR is associated with numerous gain-of-toxic function amyloid diseases called TTR amyloidoses with deposited ATTR. Today, there are many treatment options for ATTR diseases including liver transplantation, siRNA, and antisense oligonucleotides (ASOs) for modifying TTR expression as well as small molecule kinetic stabilizers to avoid tetramer dissociation and TTR misfolding and amyloid formation.^22^ There is interest in generating new and improved treatment options, but early diagnosis is even more urgent, which is the key for effective treatment. We have previously identified fluorescent ligands to report on native tetrameric and misfolded protofibrillar TTR.^18^ Ligands were based on the *trans*-stilbene resveratrol. We concluded based on pH-dependent fluorescence spectroscopy experiments that two structural analogues of *trans*-stilbenes bound with opposing directions within the T4BP. Our high-resolution crystal structures of these complexes presented in this study now confirm that the binding modes are indeed opposite, albeit contrary to our prediction. The consequences of the different ligand binding modes to TTR are important, both for fluorescence and stabilization. Our data provides the structural basis for two critical parameters to facilitate the design of TTR fluorescent ligands and inhibitors based on *trans*-stilbene scaffolds.

## Materials and Methods

### Chemicals

Chemicals were purchased from Sigma–Aldrich; columns and resins were from Cytiva. All chemicals used in the experiments were of reagent grade quality. Synthesis of ligands SB-11 and SB-14 was as previously reported.^18^

### Recombinant Expression and Purification of Wild-Type TTR and the TTR V30M Mutant

Expression and purification of TTR were carried out as described previously.^23^ The TTR wild-type and the V30M mutation were expressed and purified the same way. Briefly, *Escherichia coli* BL21 (DE3) cells were transformed with the different plasmids and grown at 37 °C. At an OD_600_ of 0.6, the temperature was lowered to 20 °C and TTR expression was induced by addition of 0.4 mM IPTG. After 18 h, cells were harvested, resuspended in 20 mM Tris-HCl, pH 8.0, 100 mM NaCl, and disrupted by sonication. After clarification the supernatant was heated to 60 °C for 30 min. Subsequently, the precipitated material was removed by centrifugation and the supernatant was filtered (0.45 μm) followed by anion exchange (Source-15Q 10/10) and size exclusion chromatography (HiPrep 16/60 Superdex75) in 10 mM Na-phosphate buffer, 100 mM KCl pH 7.6 at 20 °C. Fractions containing pure TTR were collected, pooled, and concentrated to 5.2 mg/mL, prior to flash-freezing in liquid nitrogen and storage at −80 °C until use.

### Crystallization of the TTR:Ligand Complexes

The protein was crystallized using the vapor-diffusion hanging drop method at room temperature as described previously.^23^ Briefly, purified TTR (5.2 mg/mL) was cocrystallized with either 500 μM SB-14 or SB-11 or 2 mM resveratrol (added from DMSO stock solutions (10 mM)). Drops containing 3 μL protein solution were mixed with 3 μL precipitant and equilibrated against 1 mL reservoir solution containing 1.3–1.6 M sodium citrate pH 5.5 and 3.5% *v/v* glycerol. Crystals grew to 0.1 × 0.1 × 0.4 mm after 5–7 days. Crystals were transferred into drops containing the same concentration of ligand and protein and incubated for 3 days. Crystals were cryo-protected with mother liquor supplemented with 12.5% *v/v* glycerol and ligand.

### Structure Determination

Diffraction data of the “apo”, and SB-11, SB-14, and resveratrol complex forms of TTR were collected under cryogenic conditions at BioMax (MAXIV), Sweden, at a wavelength of 0.97993 Å. Data were processed to a resolution of 1.15 Å, 1.45 Å, 1.45 Å, 1.35 Å, respectively, using XDS^24^ and AIMLESS^25^ from the CCP4 software suite.^26^ Data collection statistics are summarized in Table 2.

**Table 2:** Data Collection and Refinement Statistics

	Apo TTR	TTR + resveratrol	TTR + SB-11	TTR + SB-14
PDB code	8AWI	7Q9O	7Q9L	7Q9N
**Data collection**^a^				
Resolution	42.97–1.15 Å	38.474–1.35 Å	42.559–1.45 Å	38.087–1.45 Å
Space group	*P*2_1_22_1_	*P*2_1_22_1_	*P*2_1_22_1_	*P*2_1_22_1_
Cell parameters: *a*, *b*, *c* (Å)	42.85, 64.32, 85.86	43.07, 64.30, 85.29	43.05, 64.75, 85.05	42.55, 63.88, 85.14
α, β, χ (deg)	90.00, 90.00, 90.00	90.00, 90.00, 90.00	90.00, 90.00, 90.00	90.00, 90.00, 90.00
Completeness	98.8 (88.8)	97.9 (95.3)	100 (99.9)	99.9 (100)
Redundancy	23.1 (10.5)	6.7 (6.9)	10.5 (10.7)	13.0 (13.3)
Rmerge	0.077 (1.56)	0.038 (1.052)	0.049 (1.305)	0.053 (1.503)
Rpim	0.017 (0.486)	0.017 (0.476)	0.017 (0.431)	0.016 (0.441)
I/sigI	19.5 (1.9)	18.9 (1.7)	22.1 (2.0)	19.0 (2.0)
CC1/2	0.996 (0.670)	0.999 (0.672)	0.999 (0.799)	0.999 (0.775)
Model summary:				
Protein	2 chains: 115, 114 residues	2 chains: 113, 116 residues	2 chains: 114, 115 residues	2 chains: 114, 115 residues
Waters	240 waters	194 waters	158 waters	144 waters
	2 sodium ions	1 sodium ion	1 sodium ion	
		1 glycerol molecule	1 glycerol molecule	
		2 resveratrol molecules	2 SB-11 molecules	2 SB-14 molecules
R factor	0.14749	0.13782	0.13282	0.14243
“free” R factor	0.16577	0.17493	0.16334	0.17156
Real space correlation coeficient	0.9635	0.9604	0.9579	0.9601
Ramachandran:				
Favorable	184 (91.5%)	185 (93.0%)	187 (93.0%)	186 (92.5%)
Allowed	16 (8.0%)	14 (7.0%)	14 (7.0%)	14 (7.0%)
Acceptable	0	0	0	1 (0.5%)
Dissallowed	0	0	0	0
RMS bond lengths Z score	0.512	0.70	0.70	0.70
RMS angles Z score	0.905	0.78	0.76	0.81
G values:				
Dihedrals	–0.13	–0.20	–0.18	–0.20
Covalent	0.24	0.17	0.14	0.11
Overall	–0.02	–0.03	–0.03	–0.05
Estimated coordinate error (DPI)	0.0302	0.0478	0.0580	0.0624
Ligand validation (individual):				
Real-space R factor	N/A	0.08/0.11	0.12/0.16	0.12/0.20
Real-space correlation coeficient		0.97/0.92	0.87/0.94	0.93/0.95

a Values in parentheses are for the highest resolution shell.

Phasing was by molecular replacement using Phaser^27^ with a search model derived from the published coordinates 1F41^28^ (omitting terminal residues and a known flexible region). Ligands and solvent were placed in density after 1 to 2 initial rounds of rebuilding the protein model with COOT^29^ and refinement using REFMAC.^30^ After placing the ligands, a further 2 to 3 iterations of rebuild/REFMAC refinement were performed with ligand occupancy increasing when appropriate.

Ligand atoms were initially placed at 0.3 occupancy if clearly visible in the electron density; otherwise, occupancy was set at 0.1. Occupancy was increased in line with (i) developing density and (ii) consistency with B-factors of surrounding atoms/residues. Due to the positioning of compounds (on the special position) the maximum occupancy is 0.5.

Because of the lack of interpretable electron densities in the final map, nine N-terminal (residues 1–9) and two or three C-terminal residues were not included in the final model. A summary of the crystallographic analyses is presented in Table 2.

### Coordinates

Structure factors and coordinates of the TTR (apo), TTR:SB-11, TTR:SB-14, and TTR:resveratrol complexes have been deposited at the PDB (accession codes: 8AWI, 7Q9L, 7Q9N, and 7Q9O, respectively).

### Fibril Formation Assay

TTR (2.0 mg/mL, in PBS buffer) was preincubated with or without 2 equimolar concentrations of inhibitor (resveratrol, SB-11, or SB-14) or vehicle (DMSO). Fibril formation was induced by 10-fold dilution to a final concentration of 0.2 mg/mL in 50 mM sodium acetate buffer, 100 mM NaCl, final pH 4.4. Samples were incubated under stagnant conditions at 37 °C for 72 h in sealed 96 well plates (Corning 3880). Turbidity was measured at 400 nm after 60 s of shaking. Correction for background was performed for each sample. Fibril formation as measured by turbidity (absorbance/optical density) at 400 nm was set to 100% in the absence of inhibitor (1% DMSO vehicle) for TTR and TTR V30M, respectively. Samples were assayed in triplicate and averaged.

### Protein Thermal Stability

Samples for the thermal shift assay were prepared the same way as for fibril formation with the exception of pH 5.0 as the final pH. Samples were measured by nano-DSF using the Prometheus NT-48 (Nanotemper). After sealing the capillaries, the thermal scan was performed from 20 to 110 °C, with a ramp rate of 0.5 °C/min and recording the 330 and 350 nm intrinsic tryptophan fluorescence signal. Samples were assayed in triplicate. The inflection point of the first derivative of the Trp fluorescence monitored 350/330 nm unfolding curve was denoted as the melting temperature (*T*_m_). The mean *T*_m_ and standard deviation of three separate samples were calculated for each protein with and without ligands (1% DMSO vehicle).

## References

[ref1] Li X.; Zhang X.; Ladiwala A. R. A.; Du D.; Yadav J. K.; Tessier P. M.; Wright P. E.; Kelly J. W.; Buxbaum J. N. Mechanisms of Transthyretin Inhibition of β-Amyloid Aggregation In Vitro. J. Neurosci. 2013, 33 (50), 19423–19433. 10.1523/JNEUROSCI.2561-13.2013. 24336709 PMC3858619

[ref2] Cao Q.; Anderson D. H.; Liang W. Y.; Chou J.; Saelices L. The Inhibition of Cellular Toxicity of Amyloid-β by Dissociated Transthyretin. J. Biol. Chem. 2020, 295 (41), 14015–14024. 10.1074/jbc.RA120.013440. 32769117 PMC7549042

[ref3] Ghadami S. A.; Chia S.; Ruggeri F. S.; Meisl G.; Bemporad F.; Habchi J.; Cascella R.; Dobson C. M.; Vendruscolo M.; Knowles T. P. J.; Chiti F. Transthyretin Inhibits Primary and Secondary Nucleations of Amyloid-β Peptide Aggregation and Reduces the Toxicity of Its Oligomers. Biomacromolecules 2020, 21 (3), 1112–1125. 10.1021/acs.biomac.9b01475. 32011129 PMC7997117

[ref4] Nilsson L.; Pamrén A.; Islam T.; Brännström K.; Golchin S. A.; Pettersson N.; Iakovleva I.; Sandblad L.; Gharibyan A. L.; Olofsson A. Transthyretin Interferes with Aβ Amyloid Formation by Redirecting Oligomeric Nuclei into Non-Amyloid Aggregates. J. Mol. Biol. 2018, 430 (17), 2722–2733. 10.1016/j.jmb.2018.06.005. 29890120

[ref5] Jayaweera S. W.; Surano S.; Pettersson N.; Oskarsson E.; Lettius L.; Gharibyan A. L.; Anan I.; Olofsson A. Mechanisms of Transthyretin Inhibition of IAPP Amyloid Formation. Biomol 2021, 11 (3), 411 10.3390/biom11030411. PMC8001701 33802170

[ref6] Westermark P.; Sletten K.; Johansson B.; Cornwell G. G. Fibril in Senile Systemic Amyloidosis Is Derived from Normal Transthyretin. Proc. National Acad. Sci. 1990, 87 (7), 2843–2845. 10.1073/pnas.87.7.2843. PMC53787 2320592

[ref7] Benson M. D.; Buxbaum J. N.; Eisenberg D. S.; Merlini G.; Saraiva M. J. M.; Sekijima Y.; Sipe J. D.; Westermark P. Amyloid Nomenclature 2020: Update and Recommendations by the International Society of Amyloidosis (ISA) Nomenclature Committee. Amyloid 2020, 27 (4), 217–222. 10.1080/13506129.2020.1835263. 33100054

[ref8] Bulawa C. E.; Connelly S.; DeVit M.; Wang L.; Weigel C.; Fleming J. A.; Packman J.; Powers E. T.; Wiseman R. L.; Foss T. R.; Wilson I. A.; Kelly J. W.; Labaudinière R. Tafamidis, a Potent and Selective Transthyretin Kinetic Stabilizer That Inhibits the Amyloid Cascade. Proc. National Acad. Sci. 2012, 109 (24), 9629–9634. 10.1073/pnas.1121005109. PMC3386102 22645360

[ref9] Corazza A.; Verona G.; Waudby C. A.; Mangione P. P.; Bingham R.; Uings I.; Canetti D.; Nocerino P.; Taylor G. W.; Pepys M. B.; Christodoulou J.; Bellotti V. Binding of Monovalent and Bivalent Ligands by Transthyretin Causes Different Short- and Long-Distance Conformational Changes. J. Med. Chem. 2019, 62 (17), 8274–8283. 10.1021/acs.jmedchem.9b01037. 31393717 PMC6863598

[ref10] Mangione P. P.; Verona G.; Corazza A.; Marcoux J.; Canetti D.; Giorgetti S.; Raimondi S.; Stoppini M.; Esposito M.; Relini A.; Canale C.; Valli M.; Marchese L.; Faravelli G.; Obici L.; Hawkins P. N.; Taylor G. W.; Gillmore J. D.; Pepys M. B.; Bellotti V. Plasminogen Activation Triggers Transthyretin Amyloidogenesis in Vitro. J. Biol. Chem. 2018, 293 (37), 14192–14199. 10.1074/jbc.RA118.003990. 30018138 PMC6139548

[ref11] Berman A. Y.; Motechin R. A.; Wiesenfeld M. Y.; Holz M. K. The Therapeutic Potential of Resveratrol: A Review of Clinical Trials. Npj Precis Oncol 2017, 1 (1), 35 10.1038/s41698-017-0038-6. 28989978 PMC5630227

[ref12] Gertz M.; Nguyen G. T. T.; Fischer F.; Suenkel B.; Schlicker C.; Fränzel B.; Tomaschewski J.; Aladini F.; Becker C.; Wolters D.; Steegborn C. A Molecular Mechanism for Direct Sirtuin Activation by Resveratrol. PLoS One 2012, 7 (11), e49761 10.1371/journal.pone.0049761. 23185430 PMC3504108

[ref13] Blake C. C. F.; Geisow M. J.; Oatley S. J.; Rérat B.; Rérat C. Structure of Prealbumin: Secondary, Tertiary and Quaternary Interactions Determined by Fourier Refinement at 1.8 Å. J. Mol. Biol. 1978, 121 (3), 339–356. 10.1016/0022-2836(78)90368-6. 671542

[ref14] Florio P.; Folli C.; Cianci M.; Del Rio D.; Zanotti G.; Berni R. Transthyretin Binding Heterogeneity and Anti-Amyloidogenic Activity of Natural Polyphenols and Their Metabolites. J. Biol. Chem. 2015, 290 (50), 29769–29780. 10.1074/jbc.M115.690172. 26468275 PMC4705982

[ref15] Klabunde T.; Petrassi H. M.; Oza V. B.; Raman P.; Kelly J. W.; Sacchettini J. C. Rational Design of Potent Human Transthyretin Amyloid Disease Inhibitors. Nat. Struct. Biol. 2000, 7 (4), 312–321. 10.1038/74082. 10742177

[ref16] Hammarström P.; Jiang X.; Hurshman A. R.; Powers E. T.; Kelly J. W. Sequence-Dependent Denaturation Energetics: A Major Determinant in Amyloid Disease Diversity. Proc. National Acad. Sci. 2002, 99 (suppl_4), 16427–16432. 10.1073/pnas.202495199. PMC139904 12351683

[ref17] LeVine H.; Peter K.; Nilsson R.; Hammarström P. Bio-Nanoimaging. Part Nanoimaging Nanotechnol Aggregating Proteins Vitro Approaches 2014, 69–79. 10.1016/B978-0-12-394431-3.00007-9.

[ref18] Campos R. I.; Wu X.; Elgland M.; Konradsson P.; Hammarström P. Novel Trans-Stilbene-Based Fluorophores as Probes for Spectral Discrimination of Native and Protofibrillar Transthyretin. ACS Chem. Neurosci. 2016, 7 (7), 924–940. 10.1021/acschemneuro.6b00062. 27144293

[ref19] Johnson S. M.; Wiseman R. L.; Sekijima Y.; Green N. S.; Adamski-Werner S. L.; Kelly J. W. Native State Kinetic Stabilization as a Strategy To Ameliorate Protein Misfolding Diseases: A Focus on the Transthyretin Amyloidoses. Acc. Chem. Res. 2005, 38 (12), 911–921. 10.1021/ar020073i. 16359163

[ref20] Peterson S. A.; Klabunde T.; Lashuel H. A.; Purkey H.; Sacchettini J. C.; Kelly J. W. Inhibiting Transthyretin Conformational Changes That Lead to Amyloid Fibril Formation. Proc. National Acad. Sci. 1998, 95 (22), 12956–12960. 10.1073/pnas.95.22.12956. PMC23669 9789022

[ref21] Petrassi H. M.; Klabunde T.; Sacchettini J.; Kelly J. W. Structure-Based Design of N-Phenyl Phenoxazine Transthyretin Amyloid Fibril Inhibitors. J. Am. Chem. Soc. 2000, 122 (10), 2178–2192. 10.1021/ja993309v.

[ref22] Burton A.; Castaño A.; Bruno M.; Riley S.; Schumacher J.; Sultan M. B.; See Tai S.; Judge D. P.; Patel J. K.; Kelly J. W. Drug Discovery and Development in Rare Diseases: Taking a Closer Look at the Tafamidis Story. Drug Des Dev Ther 2021, 15, 1225–1243. 10.2147/DDDT.S289772. PMC7987260 33776421

[ref23] Iakovleva I.; Begum A.; Pokrzywa M.; Walfridsson M.; Sauer-Eriksson A. E.; Olofsson A. The Flavonoid Luteolin, but Not Luteolin-7-O-Glucoside, Prevents a Transthyretin Mediated Toxic Response. PLoS One 2015, 10 (5), e0128222 10.1371/journal.pone.0128222. 26020516 PMC4447256

[ref24] Kabsch W. XDS. Acta Crystallogr. D Biol. Crystallogr. 2010, 66 (2), 125–132. 10.1107/S0907444909047337. 20124692 PMC2815665

[ref25] Evans P. R.; Murshudov G. N. How Good Are My Data and What Is the Resolution?. Acta Crystallogr. D Biol. Crystallogr. 2013, 69 (7), 1204–1214. 10.1107/S0907444913000061. 23793146 PMC3689523

[ref26] Winn M. D.; Ballard C. C.; Cowtan K. D.; Dodson E. J.; Emsley P.; Evans P. R.; Keegan R. M.; Krissinel E. B.; Leslie A. G. W.; McCoy A.; McNicholas S. J.; Murshudov G. N.; Pannu N. S.; Potterton E. A.; Powell H. R.; Read R. J.; Vagin A.; Wilson K. S. Overview of the CCP4 Suite and Current Developments. Acta Crystallogr. Sect D Biological Crystallogr. 2011, 67 (4), 235–242. 10.1107/S0907444910045749. PMC3069738 21460441

[ref27] McCoy A. J.; Grosse-Kunstleve R. W.; Adams P. D.; Winn M.; Storoni L. C.; Read R. J. Phaser Crystallographic Software. J. Appl. Crystallogr. 2007, 40 (4), 658–674. 10.1107/S0021889807021206. 19461840 PMC2483472

[ref28] Hörnberg A.; Eneqvist T.; Olofsson A.; Lundgren E.; Sauer-Eriksson A. E. A Comparative Analysis of 23 Structures of the Amyloidogenic Protein Transthyretin11Edited by F. Cohen. J. Mol. Biol. 2000, 302 (3), 649–669. 10.1006/jmbi.2000.4078. 10986125

[ref29] Emsley P.; Cowtan K. Coot: Model-Building Tools for Molecular Graphics. Acta Crystallogr. D Biol. Crystallogr. 2004, 60 (12), 2126–2132. 10.1107/S0907444904019158. 15572765

[ref30] Murshudov G. N.; Skubák P.; Lebedev A. A.; Pannu N. S.; Steiner R. A.; Nicholls R. A.; Winn M. D.; Long F.; Vagin A. A. REFMAC5 for the Refinement of Macromolecular Crystal Structures. Acta Crystallogr. D Biol. Crystallogr. 2011, 67 (4), 355–367. 10.1107/S0907444911001314. 21460454 PMC3069751

